# Synthetic Circular RNA for microRNA-1269a Suppresses Tumor Progression in Oral Squamous Cell Carcinoma

**DOI:** 10.3390/cancers16061242

**Published:** 2024-03-21

**Authors:** Atsushi Kasamatsu, Ryunosuke Nozaki, Kohei Kawasaki, Tomoaki Saito, Chikashi Minemura, Naohiko Seki, Joel Moss, Katsuhiro Uzawa

**Affiliations:** 1 Department of Dentistry and Oral-Maxillofacial Surgery, Chiba University Hospital, 1-8-1 Inohana, Chuo-ku, Chiba-shi 260-8677, Chiba, Japan; nozaki.r@chiba-u.jp; 2 Department of Oral Science, Graduate School of Medicine, Chiba University, 1-8-1 Inohana, Chuo-ku, Chiba-shi 260-8670, Chiba, Japan; kou0802hei@gmail.com (K.K.); tomoakisaito@chiba-u.jp (T.S.); 3 Department of Oral and Maxillofacial Surgery, National Defense Medical College Hospital, Tokorozawa 359-8513, Saitama, Japan; minichika.cm@gmail.com; 4 Department of Functional Genomics, Graduate School of Medicine, Chiba University, Chiba-shi 260-8670, Chiba, Japan; naoseki@faculty.chiba-u.jp; 5 Critical Care Medicine and Pulmonary Branch, National Heart, Lung, and Blood Institute, National Institutes of Health, Bethesda, MD 20892-1590, USA; mossj@nhlbi.nih.gov

**Keywords:** circRNA, artificial synthetic circRNA, microRNA, miR-1269a, phospholipase C gamma 2, squamous cell carcinoma

## Abstract

**Simple Summary:**

Circular RNAs (circRNAs) are a new class of unique RNAs that have single-stranded, covalently closed, and continuous loop structures, which confer stability and resistance to endonuclease-mediated degradation that in turn affords substantially longer circulatory half-lives relative to linear RNAs. In this study, we established a novel synthetic circRNA that carries miR-1269a binding sequences and investigated its potential application as a therapeutic tool to slow cancer progression. Briefly, phospholipase C gamma 2 (PLCG2), which is related to oral squamous cell carcinoma (OSCC) clinical stage and overall survival, was affected by the circRNA-1269a/miR-1269a axis. Our confirmation that circRNA-1269a/miR-1269a/PLCG2 can inhibit OSCC proliferation and migration by promoting apoptosis in OSCC cells in vitro indicates that PLCG2 overexpression via the circRNA1269a/miR-1269a axis may be a valuable tool for controlling OSCC growth. Therefore, these data suggest that circRNA-1269a/miR-1269a could act as a potential therapeutic axis for OSCCs.

**Abstract:**

microRNAs (miRs) function in cancer progression as post-transcriptional regulators. We previously reported that endogenous circular RNAs (circRNAs) function as efficient miR sponges and could act as novel gene regulators in oral squamous cell carcinoma (OSCC). In this study, we carried out cellular and luciferase reporter assays to examine competitive inhibition of miR-1269a, which is upregulated expression in several cancers, by circRNA-1269a, a synthetic circRNA that contains miR-1269a binding sequences. We also used data-independent acquisition (DIA) proteomics and in silico analyses to determine how circRNA-1269a treatment affects molecules downstream of miR-1269a. First, we confirmed the circularization of the linear miR-1269a binding site sequence using RT-PCR with divergent/convergent primers and direct sequencing of the head-to-tail circRNA junction point. In luciferase reporter and cellular functional assays, circRNA-1269a significantly reduced miR-1269a function, leading to a significant decrease in cell proliferation and migration. DIA proteomics and gene set enrichment analysis of OSCC cells treated with circRNA-1269a indicated high differential expression for 284 proteins that were mainly enriched in apoptosis pathways. In particular, phospholipase C gamma 2 (PLCG2), which is related to OSCC clinical stage and overall survival, was affected by the circRNA-1269a/miR-1269a axis. Taken together, synthetic circRNA-1269a inhibits tumor progression via miR-1269a and its downstream targets, indicating that artificial circRNAs could represent an effective OSCC therapeutic.

## 1. Introduction

microRNAs (miRs) are small non-coding RNAs that modulate translational repression or degradation of mRNA molecules by binding to 3′ untranslated regions of target mRNAs [1]. miRs have been extensively understood in critical biological processes including tumor proliferation, apoptosis, and differentiation. Aberrant miR expression is linked closely to the initiation and progression of numerous cancers [2]. Therefore, disturbance of miRs can act as oncogenes (onco-miRs) or tumor suppressor genes (ts-miRs) and play critical roles in tumorigenesis and progression [2,3,4,5].

Circular RNAs (circRNAs) are a novel class of unique RNAs, characterized by single-stranded, covalently closed, and continuous loop structures [6]. circRNAs are produced by backsplicing or head-to-tail splicing of linear RNAs [7] to form covalently closed loop structures without 5′−3′ polarity or a polyadenylated tail [6,8,9,10,11]. The loop structures of circRNAs confer stability and resistance to endonuclease-mediated degradation that in turn affords substantially longer circulatory half-lives relative to linear RNAs [12]. Endogenous circRNAs contain selectively conserved miR binding sites and thereby can function as efficient miR sponges that counteract miR activity [12,13,14,15]. Although recent research on circRNAs provided promising new insights into miR regulation [12,15], the construction and application of artificial synthetic circRNAs targeting specific onco-miRs remains limited.

miR-1269a is one of the most well-characterized onco-miRs and is overexpressed in several cancers [16,17,18,19]. Elevated expression of miR-1269a has been implicated in critical cancer-related processes, including proliferation, invasion, and apoptosis [17,18,19]. In the present study, we established a novel synthetic circRNA that carries miR-1269a binding sequences and investigated its potential application as a therapeutic tool to slow cancer progression.

## 2. Materials and Methods

### 2.1. Cell Culture

Human OSCC cell lines (Sa3, HSC3, HSC2, HSC4, and SAS) were purchased from the Japanese Collection of Research Bioresources Cell Bank (Ibaraki, Japan) and the RIKEN BioResource Center (Tsukuba, Japan). Short tandem repeat analysis by PCR amplification was performed for cell authentication. OSCC cells were cultured at 37 °C with 5% CO_2_.

### 2.2. Differential Expression of miR-1269a in the Cancer Genome Atlas (TCGA)

We used TCGA head and neck squamous cell carcinoma (HNSC) clinical data obtained from cBioportal (https://www.cbioportal.org, accessed on 10 April 2020). Among TCGA-HNSC, we selected OSCC samples whose primary sites were the alveolar ridge, buccal mucosa, floor of mouth, hard palate, lip, and tongue. A total of 297 OSCCs and 30 normal samples for which miR-1269a sequence data were available were retrieved from the TCGA data portal. Analysis of differential miR-1269a expression was performed according to the previous study [16].

### 2.3. qRT-PCR Analysis of miR-1269a Expression

Total RNA from the OSCC cell lines Sa3, HSC3, HSC2, HSC4, and SAS was extracted using a microRNA purification kit (Thermo Fisher Scientific, Waltham, MA, USA) and reverse-transcribed. Expression levels of miR-1269a were measured by qRT-PCR according to the previous study [16]. All qRT-PCR assays were performed in triplicate.

### 2.4. Luciferase Reporter Assays

**Figure 1:**
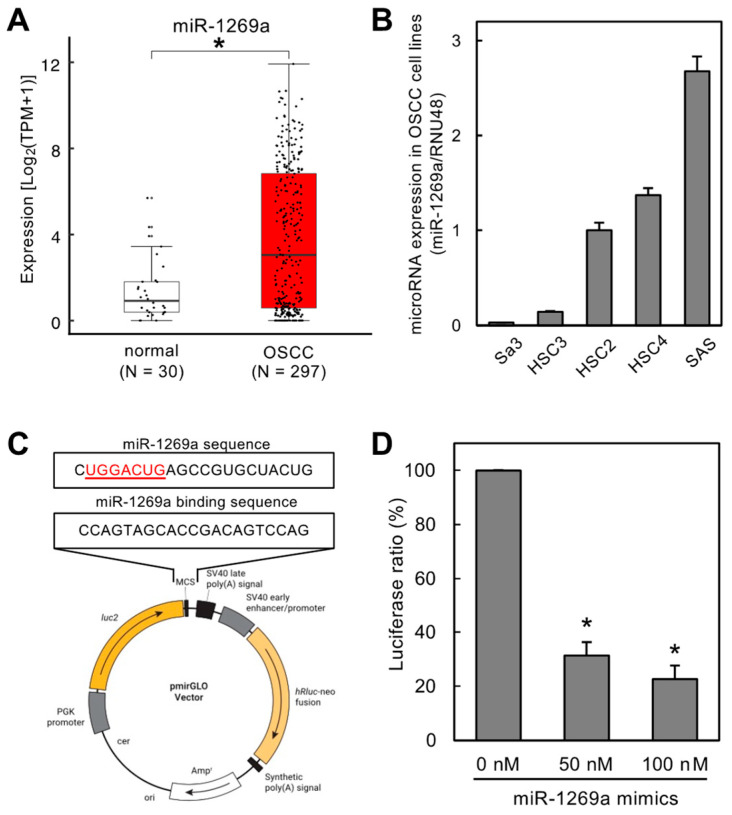
Validation of a specific sequence to maximize the sponge function toward miR-1269a. (**A**) TCGA dataset showing significantly upregulated expression of miR-1269a in OSCC tissues (N = 297) compared to healthy counterparts (N = 30) (* *p* < 0.05). (**B**) miR-1269a expression levels measured by qRT-PCR to define the optimal OSCC cell line for further functional assays. miR-1269a expression in SAS cells was higher than in the other OSCC cell lines tested. (**C**) Construction of a luciferase vector containing the specific sequence needed for miR-1269a sponge function. (**D**) Luciferase assay to confirm Luc-1269a sponge function toward miR-1269a. Luciferase activity after treatment with miR-1269a mimics was significantly lower than that for mock-treated control cells (* *p* < 0.05).

A luciferase reporter vector (pmirGLO, Promega, Madison, WI, USA) containing the miR-1269a binding site (CCAGTAGCACCGACAGTCCAG) was constructed using *Pme*I and *Xba*I restriction enzymes (Figure 1C, Luc-1269a, Takara Bio, Inc., Kusatsu, Shiga, Japan). HEK293 cells (1.0 × 10^4^ cells) were seeded in 96-well plates and co-transfected with Luc-1269a and either 50 or 100 nM of miR-1269a mimics. At 24 h after transfection, luciferase reporter assays were performed according to the previous study [16].

### 2.5. Design and Synthesis of miR-1269a-Specific circRNA (circRNA-1269a)

circRNA-1269a including two miR-1269a binding sites (2X circRNA-1269a) was synthesized using two annealing oligomers: 5′-GATTCCTTGGCCAGTAGCACCGACAGTCCAGCCAACCAGTAGCACCGACAGTCCAG-3′ (sense) and 5′-CTGGACTGTCGGTGCTACTGGTTGGCTGGACTGTCGGTGCTACTGGCCAAGGAATC-3′ (antisense). The product was generated by adding a dA to the 3′ end with a Mighty TA-cloning Reagent set (Takara Bio Inc). To synthesize 4X circRNA-1269a, one cycle of overlap PCR was performed to generate a double-stranded DNA PCR product for synthesis. The overlapping complementary 3′ ends allowing the two oligomers (5′-GATTCCTTGGCCAGTAGCACCGACAGTCCAGCCAACCAGTAGCACCGACAGTCCAGCCAACCAGTAGCACCGACAGTCCAGAATGCTGAT-3′ (sense) and 5′-CTGGACTGTCGGTGCTACTGGCCAACTGGACTGTCGGTGCTACTGGCCAAATCAGCATTCTGGACTGTCGG-3′ (antisense)) to anneal to one another after heating were used as template DNA for PCR to produce the full-length product, which was then cloned into the pCR™2.1-TOPO™ vector (Thermo Fisher Scientific) and transformed into *E. coli*. Plasmid DNA was extracted and digested with *Bam*HI (New England Biolabs, Ipswich, MA, USA). Large quantities of 5′-monophosphorylated linear RNA were synthesized using T7 RNA polymerase (New England Biolabs). After purification, the linear RNA was treated first with calf intestinal alkaline phosphatase (New England Biolabs) and then with T4 polynucleotide kinase (New England Biolabs). Finally, the linear RNA was circularized using T4 RNA ligase (New England Biolabs).

### 2.6. Confirmation of circRNA-1269a Synthesis

To verify the closed-loop structure of circRNA-1269a, reverse-transcribed cDNA was amplified using a TopTaq Master Mix kit (Qiagen, Germantown, MD, USA) with divergent or convergent primers. After reverse transcription, divergent primers (5′-AGGGCGAATTCCAGCACACTGGC-3′ (sense) and 5′-CCAAGGAATCAAGGGCGAATTCTGCAGA-3′ (antisense)) were used to amplify a DNA PCR fragment containing the head-to-tail circRNA junction point. Convergent primers (5′-GCGAATTGGGCCCTCTAGATGCATGCTC-3′ (sense) and 5′-CACTAGTAACGGCCGCCAGTGTGCTG-3′ (antisense)) were used to amplify a DNA PCR fragment containing 2 or 4 repeats of the miR-1269a binding sites. PCR products were then cloned and sequenced.

### 2.7. Transfection of circRNA-1269a

SAS cells were seeded in 6-well plates at 2.0 × 10^5^ cells/well prior to transfection with 10 nM circRNA-1269a (2X or 4X) using Lipofectamine 3000 (Thermo Fisher Scientific), according to the manufacturer’s instructions. The cells were incubated for 24–96 h and collected for subsequent functional experiments.

### 2.8. Cell Proliferation Assay

Cellular proliferation assays to examine how circRNA-1269a affects cellular growth were conducted using circRNA-1269a-treated cells seeded in 60 mm dishes at 1.0 × 10^4^ viable cells/dish. The cells were counted using a Luna^TM^ Automated Cell Counter (Logos Biosystems, Annandale, VA, USA) at 24 h intervals from 24 to 120 h.

### 2.9. Migration Assay

To investigate how circRNA-1269a affects cellular migration, transfected cells were plated in 6-well culture plates in DMEM containing 10% FBS and cultured until confluence was achieved. A wound was created in the middle of each plate using a 200 µL micropipette tip. The plates were then incubated at 37 °C, 5% CO_2_ in serum-free medium. Migration was visualized by measuring the area of the wound every 12 h using Lenaraf220b free software (http://www.vector.co.jp/soft/dl/win95/art/se312811.html (accessed on 10 April 2020)).

### 2.10. Proteomic Analysis of the Effects of circRNA-1269a Treatment

After circRNA treatment, cells were harvested with a cell scraper, resuspended in 30 mL PBS, collected with two rounds of centrifugation, and stored at −80 °C until use. Data-independent acquisition (DIA) proteomic analysis was performed by Kazusa Genome Technologies, Inc. (Kisarazu, Japan) according to a previously reported method [20]. The threshold to define altered protein expression was ≥2.0-fold change and *p* < 0.05 (Welch’s *t*-test) between the treated and untreated groups. The expression intensity values for proteins with significant differential expression were obtained using a fold-change cutoff ≥ 2.0 or ≤0.5 (*p* < 0.05) and visualized by volcano plots [21].

### 2.11. Gene Set Enrichment Analysis (GSEA)

GSEA was used to analyze the molecular pathways related to proteins with upregulated expression after circRNA-1269a treatment. The protein lists were uploaded into GSEA software (https://www.gsea-msigdb.org/gsea/login.jsp (accessed on 1 May 2022)) [22,23], and the Hallmark gene set in The Molecular Signatures Database was applied [23,24].

### 2.12. Identification of miR-1269a Targets

The miR-1269a target genes were predicted based on the TargetScan Human database (Release 7.2, http://www.targetscan.org/vert_72/; accessed on 1 August 2019). We then compared the miR-1269a target genes and upregulated proteins to find the common targets of miR-1269a. TCGA clinical data from cBioPortal (https://www.cbioportal.org; data downloaded on 12 June 2021) were used for Kaplan–Meier analyses of overall survival for common targets of miR-1269a. Gene expression grouping data for each gene were collected from OncoLnc (http://www.oncolnc.org/; accessed on 18 August 2021). 

### 2.13. Analysis of PLCG2 in OSCC Patients

The clinical significance of PLCG2 was investigated using OSCC data among TCGA-HNSC (https://tcga-data.nci.nih.gov/tcga/; accessed on 12 June 2021; OSCC, 314 cases; Normal, 30 cases). Clinical parameters and gene expression data were obtained from cBioPortal (http://www.cbioportal.org/; accessed on 13 July 2019) [25,26] and OncoLnc (http://www.oncolnc.org/; accessed on 1 August 2019) [27]. Monovariate and multivariate analyses were also performed according to the previous study [16]. Multivariate analyses were performed by JMP Pro 15.0.0 (SAS Institute Inc., Cary, NC, USA).

### 2.14. Statistical Analyses

Welch’s *t*-test assessed the comparisons between the treated and untreated groups. Dunnett’s test assessed differences between multiple groups were compared with the control group. SPSS 20.0 was used for all statistical analyses, and *p* < 0.05 was defined as a statistical significance cutoff. All experiments were repeated at least three times. Means ± SEM are displayed as representative values in the figures.

## 3. Results

### 3.1. Expression Levels of miR-1269a in OSCC Patients in the TCGA Dataset and OSCC Cell Lines

The TCGA dataset showed that miR-1269a expression was significantly upregulated in OSCC tissues (N = 297) compared with normal counterparts (N = 30) (Figure 1A; *p* < 0.05). We also used qRT-PCR to measure miR-1269a expression levels in different OSCC cell lines to determine the optimal line for subsequent functional assays (Figure 1B). The miR-1269a levels were highest in SAS cells, and this line was used for further studies.

### 3.2. Validation of a Specific Sequence for miR-1269a Sponge Function

We constructed a luciferase vector (Luc-1269a) containing a specific sequence that has a sponge function toward miR-1269a (Figure 1C). We confirmed this sponge function using HEK293 cells co-transfected with Luc-1269a and miR-1269a mimics. Luciferase activity of Luc-1269a co-transfected with miR-1269a mimics (50 or 100 nM) was significantly lower than that for control cells (0 nM), indicating that the specific sequence on Luc-1269a has a sponge function for miR-1269a (Figure 1D, *p* < 0.05).

### 3.3. Construction and Verification of circRNA-1269a

**Figure 2:**
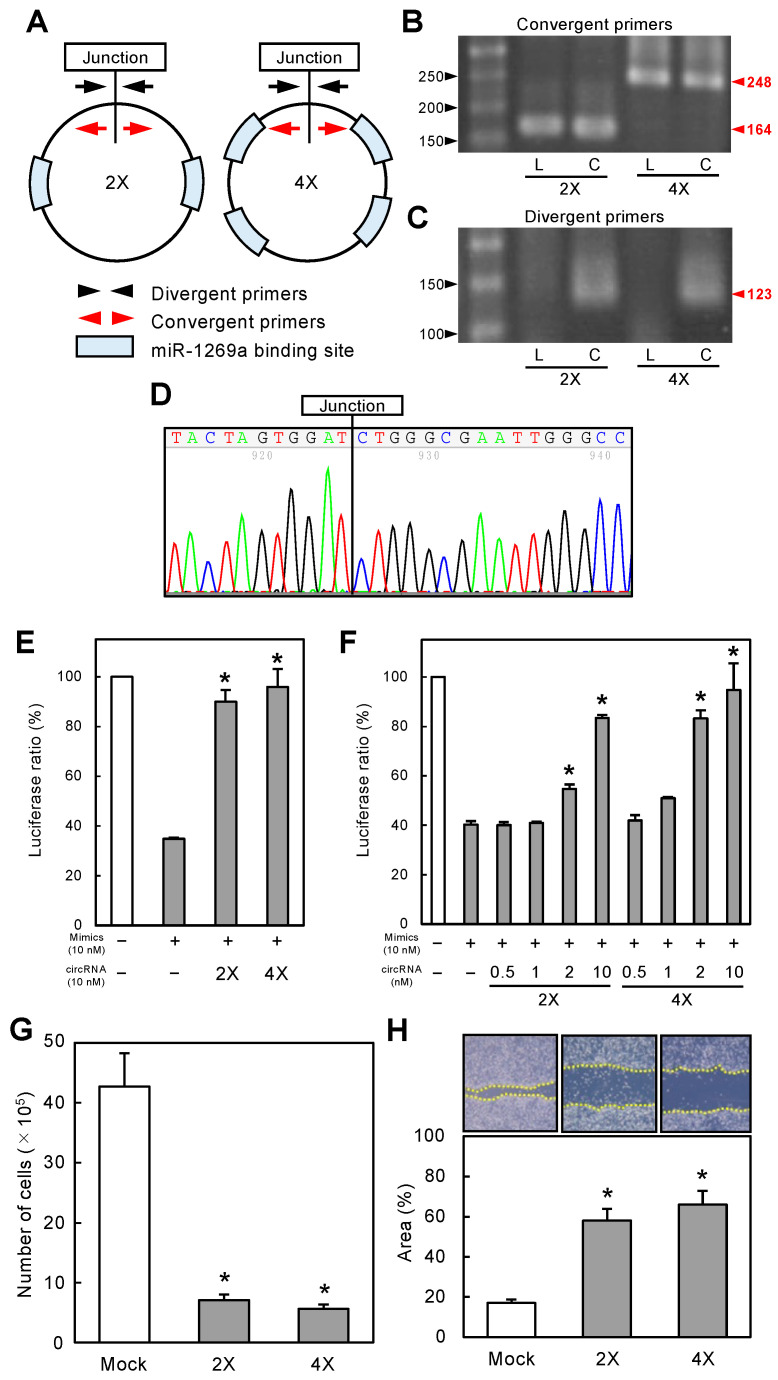
Construction of circRNA-1269a and its functional effects. (**A**) circRNA-1269a constructs, where 2X and 4X contain 2 and 4 repeats of miR-1269a binding sites, respectively. (**B**) Both linearRNA-1269a (*L*) and circRNA-1269a (**C**) yielded expected products when using convergent RT-PCR primers (2X, 164 bp; 4X, 248 bp). (**C**) Divergent primers generate the expected products in circRNA-1269a (*C*) (123 bp). (**D**) Sanger sequencing of the PCR products using divergent primers confirmed that circRNA-1269a has the correct end-to-end ligation junction. (**E**) Luciferase activity (Luc-1269a) in cells co-transfected with miR-1269a mimics and circRNA-1269a is significantly higher than for cells transfected with only miR-1269a mimics (*, *p* < 0.05). (**F**) Dose-dependent sponge function of circRNA-1269a. As the circRNA-1269a concentration increases, stimulatory effects on luciferase activity strengthen, with the maximal effect seen at 10 nM, the highest concentration tested (*, *p* < 0.05). (**G**) Cellular proliferation of circRNA-1269a-treated cells is significantly decreased relative to mock-treated cells 72 h after treatment (*, *p* < 0.05). (**H**) The wound area decreased significantly in circRNA-1269a-treated cells after 12 h compared with that for mock-treated cells (*, *p* < 0.05).

RNA circularization was performed by enzymatic ligation using synthetic linear RNAs containing two or four miR-1269a binding sites (linearRNA-1269a) termed 2X and 4X miR-1269a, respectively (Figure 2A). After synthesis, RT-PCR and sequencing were used to verify the correct construction of circRNA-1269a. PCR products obtained using divergent primers were amplified from the end-to-end junction regions, whereas PCR products using convergent primers contained two or four miR-1269a binding sites (Figure 2A). RT-PCR results showed that both circRNA-1269a and linearRNA-1269a yielded the expected products when amplified using convergent primers (2X, 164 bp; 4X, 248 bp; Figure 2B and Figure S1). In contrast, divergent primers generated the expected products only for circRNA-1269a (Figure 2C and Figure S1; 123 bp). Moreover, Sanger sequencing of the PCR products using divergent primers confirmed the correct sequence in the end-to-end ligation junction of circRNA-1269a (Figure 2D).

### 3.4. Functional Effects of Synthetic miR-1269a-Specific circRNA

To examine the functional capability of circRNA-1269a, normalized luciferase reporter assays (Firefly luciferase/Renilla luciferase activities) were conducted after co-transfection of cells with Luc-1269a, circRNA-1269a, and miR-1269a mimics. Successful activity of circRNA-1269a would involve a sponge function that counteracts the inhibitory effects of the miR-1269a mimics. Thus, we predicted that circRNA-1269a would preserve the luciferase activity of Luc-1269a by competitively binding to miR-1269a. The luciferase activity of Luc-1269a in cells co-transfected with miR-1269a mimics and circRNA-1269a was indeed significantly greater than in cells transfected with only miR-1269a mimics (Figure 2E, *p* < 0.05). Moreover, these effects were dose-dependent. As the concentration of circRNA-1269a increased, the stimulatory effects on luciferase activity became stronger with a maximal effect at the highest concentration of 10 nM (Figure 2F, *p* < 0.05). Thus, we used 10 nM circRNA-1269a for subsequent functional studies.

### 3.5. circRNA-1269a Inhibits Proliferation and Migration of OSCC Cells

To evaluate the effect of circRNA-1269a on cellular growth, we performed a cellular proliferation assay. We observed a significant decrease in proliferation of circRNA-1269a-treated cells compared with mock-treated cells (72 h after treatment; Figure 2G; *p* < 0.05). The migration assay showed that the area of a wound created with a micropipette tip decreased significantly (*p* < 0.05) in circRNA-1269a-treated cells after 12 h compared with mock-treated cells (Figure 2H, *p* < 0.05).

### 3.6. Proteomic Analysis of circRNA-1269a-Treated OSCC Cells

**Figure 3:**
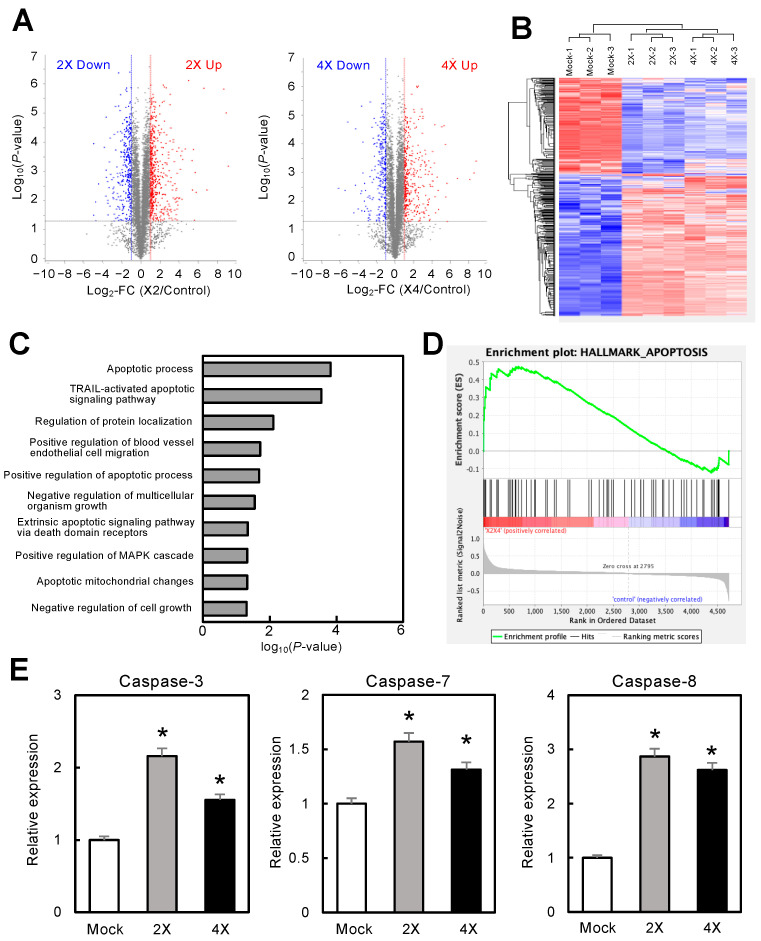
Proteomic analysis of circRNA-1269a-treated OSCC cells. (**A**) Volcano plots of protein expression data from DIA proteomic analysis. The X- and Y-axis show the log_2_-fold change (FC) and log_10_ (*p*-value), respectively. Blue points represent downregulated proteins with an absolute log_2_-FC < −2.0. Red points represent upregulated proteins with an absolute log_2_-FC > 2.0. (**B**) Unsupervised hierarchical clustering of all expressed proteins shows a clear differential pattern for circRNA-1269a treatment. (**C**,**D**) GSEA using TCGA-HNSC RNA-seq data reveals that “apoptosis” is the most enriched pathway in the circRNA-1269a-treated group. (**E**) Overexpression of the apoptosis-related proteins caspase-3, -7, and -8 was observed after treatment with circRNA-1269a (*, *p* < 0.05).

miRs exert at least some effects on target mRNAs by altering their translation into proteins. To elucidate the mechanisms of circRNA-1269a, we performed protein expression analyses of DIA proteomics data collected from circRNA-1269a-treated and untreated (mock) cells. We identified 4729 individual proteins. Volcano plots of data for circRNA-1269a-treated cells show proteins having expression that was significantly (*p* < 0.05) upregulated (2X, 542 proteins; 4X, 458 proteins) or downregulated (2X, 351 proteins; 4X, 289 proteins) by more than two-fold (Figure 3A). Unsupervised hierarchical clustering of all expressed proteins showed a clear differential protein expression pattern for cells with circRNA-1269a treatment (Figure 3B). To identify the molecular pathways in OSCC cells that were affected by circRNA-1269a treatment, we performed gene set enrichment analysis (GSEA) using TCGA-HNSC RNA-seq data. GSEA analysis revealed that “apoptosis”, and particularly the apoptotic proteins caspase-3, -7, and -8, was the most enriched pathway in the circRNA-1269a-treated cells (Figure 3C–E; *p* < 0.05). These results suggested that circRNA-1269a treatment contributes to an antitumor effect toward OSCC via an apoptosis pathway.

### 3.7. Function of the circRNA-1269a Target Protein, PLCG2, in OSCC Patients

**Figure 4:**
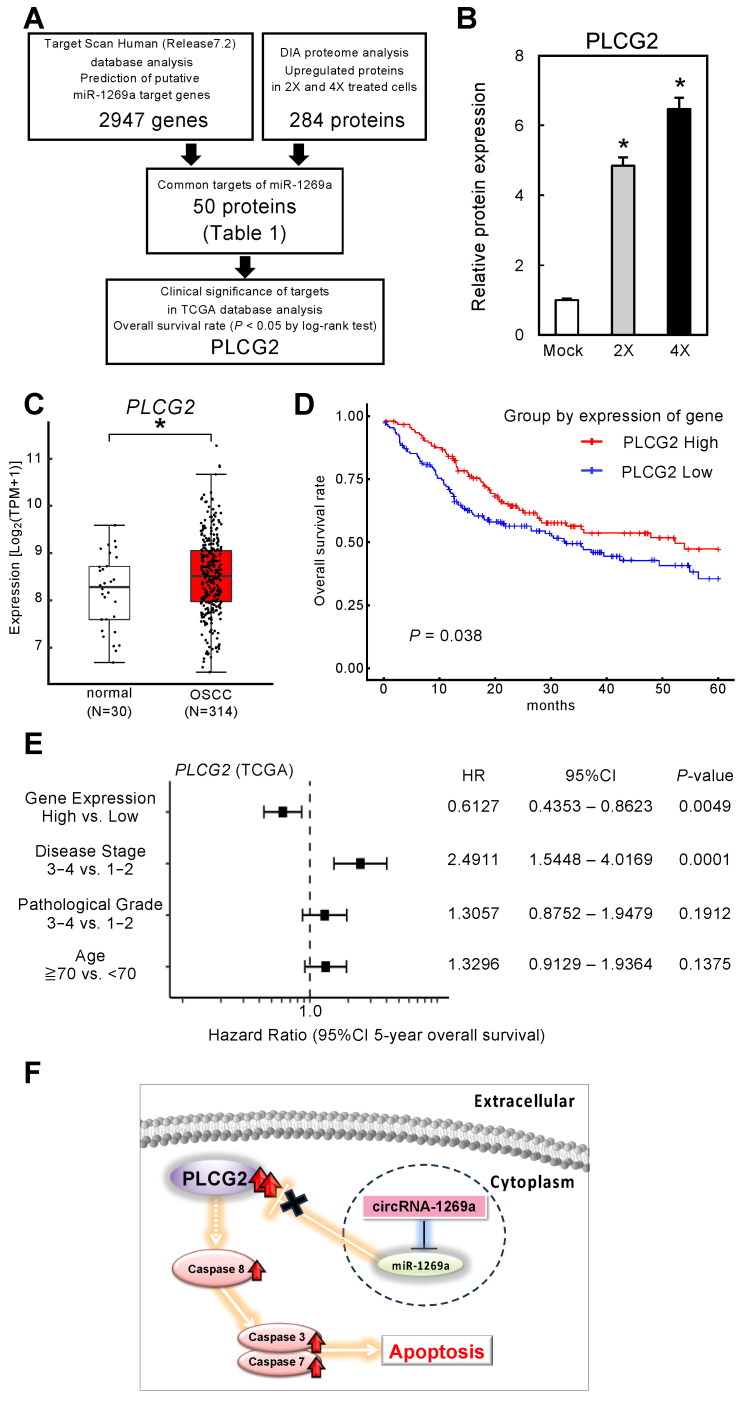
Function of the circRNA-1269a target, PLCG2, in OSCC patients. (**A**) A total of 2947 genes having a putative miR-1269a binding site were identified in a search of the TargetScan Human database (Release 7.2). In cells treated with 2X or 4X circRNA-1269a, 284 proteins were identified in a DIA proteomic analysis, and of these, 50 proteins were also identified in the TargetScan database search. These 50 proteins were assessed based on overall survival rate and expression levels in the TCGA database. PLCG2 was identified as a target protein of circRNA-1269a. (**B**) PLCG2 protein expression after circRNA-1269a treatment (*, *p* < 0.05). (**C**) Clinical significance of PLCG2 in OSCC clinical specimens determined by TCGA analysis (*, *p* < 0.05). (**D**) Kaplan–Meier curves of 5-year overall survival frequencies according to PLCG2 expression. (**E**) Forest plot showing results of multivariate analysis of PLCG2 identified by analysis of the TCGA-HNSC dataset (HR: hazard ratio; CI: confidence interval). (**F**) Schematic representation role of the circRNA-1269a/miR-1269a/PLCG2 axis that leads to apoptosis.

The strategy used to identify miR-1269a targets was summarized in Figure 4A. Based on a search of the TargetScan Human database (Release 7.2) for common putative target proteins, we found 2947 genes that had putative miR-1269a binding sites (Figure 4A). Furthermore, DIA proteomic analysis found 284 proteins containing putative miR-1269a binding sites in 2X and 4X circRNA-1269a-treated cells. A total of 50 putative target proteins were identified in both the proteomic analysis and the TargetScan Human database search (Table 1). We assessed these 50 proteins in terms of information for overall survival rate and expression levels in the TCGA database. We selected PLCG2 as a downstream target of circRNA-1269a (Figure 4A). PLCG2 was overexpressed in OSCC cells treated with circRNA-1269a (Figure 4B), suggesting that the circRNA-1269a/miR-1269a/PLCG2 axis is an important pathway in OSCC cells. In addition, the TCGA dataset showed that *PLCG2* gene expression is significantly upregulated in OSCC tissues (N = 314) compared with healthy counterparts (N = 30) (Figure 4C; *p* < 0.05). Cox proportional hazards regression analysis of PLCG2 expression was performed for 5-year overall survival rates (Figure 4D), *PLCG2* gene expression level, disease stage, pathological grade, and patient age. The multivariate analysis showed that PLCG2 expression level was an independent prognostic factor (HR 0.6127, *p* < 0.05; Figure 4E). These results indicated that PLCG2 has a tumor-suppressive function related to molecular pathogenesis and clinical prognosis in OSCC patients (Figure 4F).

## 4. Discussion

OSCC can have a poor prognosis and a high recurrence rate [28,29,30,31]. Although research on OSCC has advanced substantially, the five-year overall survival rate for this cancer remains below 50% [32]. Therefore, novel therapeutic targets for OSCC that act at a molecular level are needed. Numerous endogenous circRNAs are gaining increasing attention in cancers [13,33,34,35]. Several reports [13,33,34,35], including our previous study [13], showed that circRNAs are involved in the progression, metastasis, and multidrug resistance of cancers, suggesting that circRNAs could be diagnostic and/or therapeutic targets for multiple cancers. In addition, circRNAs have two characteristics, namely multiple miR binding sites that inhibit the function of the target miR and higher resistance to nuclease degradation relative to linear RNAs. Therefore, we hypothesized that synthetic circRNAs, which have been designed to function as exogenous miR inhibitors, would show promise for molecular cancer therapy [36].

In the present study, we selected miR-1269a as the target of newly developed synthetic circRNAs. Our previous data revealed that miR-1269a is upregulated in head and neck SCCs [16]. miR-1269a also promotes tumor progression and acts as an onco-miR in lung and gastric cancers, leading to the inhibition of apoptosis [17,18,19]. Based on our previously determined specific binding sequence for miR-1269a (Figure 1), we constructed an artificial synthetic circRNA that includes the miR-1269a binding sequence (circRNA-1269a) and that was circularized by novel enzymatic ligation (Figure 2). We confirmed that circRNA-1269a indeed has a sponge function for miR-1269a (Figure 2) and investigated downstream functions of miR-1269a in OSCC cells treated with circRNA-1269a. Our data demonstrated that circRNA-1269a inhibited cell proliferation and migration of the OSCC cell line by competitive inhibition of miR-1269a activity (Figure 2). Gene Ontology and GSEA of DIA proteomics data showed that circRNA-1269a treatment promotes apoptosis (Figure 3). In addition, our search of the TargetScan database identified PLCG2, which is involved in various biological processes including cell proliferation, cell migration, and apoptosis, as a target molecule of circRNA1269a/miR-1269a (Figure 4). Similar to previous reports showing that overexpression of PLCG2 occurs in gastric cancer, lymphoma, and hepatocytes [37,38,39,40] and that increased phosphorylation of p38 and JNK leads to apoptosis [40], our data here demonstrated overexpression of PLCG2 and marked enhancement of caspase-3, -7, and -8 expression in OSCC cells treated with circRNA-1269a, thus confirming that PLCG2 may enhance apoptosis in OSCCs. p38 and JNK signaling pathways are two important intracellular signaling pathways that are known to be tightly associated with apoptosis [41,42]. Caspase-8 is the most upstream component in the caspase cascade [43,44]. Meanwhile, caspase-3 and -7 are downstream effectors of the two apoptotic pathways and cleave various cytoplasmic and nuclear substrates including DNA fragmentation factor and DNA-dependent protein kinase [45,46].

Although a circRNA delivery system for OSCC regions is required, these data suggest that circRNA-1269a/miR-1269a could act as a potential therapeutic axis for OSCCs. We also demonstrated that circRNA-1269a-induced activation of PLCG2 and its downstream pathways may be involved in these biological processes. Our confirmation that circRNA-1269a/miR-1269a/PLCG2 can inhibit OSCC proliferation and migration by promoting apoptosis in OSCC cells in vitro indicates that PLCG2 overexpression via the circRNA1269a/miR-1269a axis may be a valuable tool for controlling OSCC growth (Figure 4F). These findings further establish that the design and construction of efficient artificial synthetic circRNAs represents a novel strategy to achieve miR loss-of-function in molecular cancer therapeutics and could have potential future therapeutic applications for cancers.

## 5. Conclusions

These results suggest that synthetic circRNA-1269a inhibits tumor progression via effects on miR-1269a and its downstream targets, indicating that artificial circRNAs could represent an effective OSCC therapeutic.

## Figures and Tables

**Table 1:** Identification of common putative targets by TargetScan database and DIA proteomic analysis.

Protein Name	Symbol	Ave Ratio		*p*-Value
		2X	4X	
Calcium-binding and coiled-coil domain-containing protein 2	CALCOCO2	2.20	2.11	2.68 × 10^−7^
Collagen and calcium-binding EGF domain-containing protein 1	CCBE1	28.49	26.84	3.41 × 10^−5^
Cap-specific mRNA (nucleoside-2′-O-)-methyltransferase 1	CMTR1	2.64	2.19	8.67 × 10^−7^
Cytokine receptor-like factor 3	CRLF3	2.32	2.36	1.25 × 10^−4^
Deoxycytidine kinase	DCK	2.35	2.17	1.44 × 10^−5^
DCN1-like protein 5	DCUN1D5	2.20	2.04	4.82 × 10^−7^
Coagulation factor X	F10	72.09	87.02	4.06 × 10^−6^
Fibroblast growth factor 2	FGF2	2.63	2.85	5.48 × 10^−7^
Peptidyl-prolyl cis-trans isomerase FKBP4	FKBP4	2.50	2.20	1.13 × 10^−6^
Glucosamine 6-phosphate N-acetyltransferase	GNPNAT1	2.27	2.13	3.88 × 10^−5^
Interferon-induced protein with tetratricopeptide repeats 1	IFIT1	44.90	40.03	5.39 × 10^−10^
Interferon-induced protein with tetratricopeptide repeats 5	IFIT5	6.50	5.34	9.78 × 10^−8^
Interferon-related developmental regulator 1	IFRD1	2.07	2.00	4.98 × 10^−8^
Interleukin-1 receptor accessory protein-like 1	IL1RAPL1	2.98	3.30	1.53 × 10^−2^
tRNA N(3)-methylcytidine methyltransferase METTL2B	METTL2B	3.87	3.33	3.88 × 10^−6^
Neuron navigator 1	NAV1	4.05	4.22	1.68 × 10^−2^
U8 snoRNA-decapping enzyme	NUDT16	3.07	2.55	6.66 × 10^−7^
Partitioning defective 6 homolog beta	PARD6B	2.12	2.27	1.32 × 10^−7^
Protocadherin-7	PCDH7	2.04	2.15	4.26 × 10^−3^
Chronophin	PDXP	2.93	2.30	3.78 × 10^−5^
1-phosphatidylinositol 4,5-bisphosphate phosphodiesterase gamma-2	PLCG2	4.85	6.47	3.81 × 10^−3^
Pleckstrin homology domain-containing family B member 2	PLEKHB2	3.85	4.31	1.16 × 10^−4^
Phospholipid scramblase 1	PLSCR1	4.49	4.23	2.25 × 10^−7^
Purine nucleoside phosphorylase	PNP	2.44	2.37	8.05 × 10^−7^
Podocalyxin	PODXL	2.12	2.42	4.34 × 10^−5^
Amidophosphoribosyltransferase	PPAT	2.40	2.17	6.02 × 10^−7^
Proteasome assembly chaperone 1	PSMG1	2.44	2.04	1.90 × 10^−6^
Sulfhydryl oxidase 1	QSOX1	2.02	2.08	1.32 × 10^−6^
Ras-related protein Rab-43	RAB43	2.15	2.10	2.17 × 10^−7^
Serine/threonine-protein kinase RIO2	RIOK2	2.18	2.06	4.80 × 10^−7^
GTP-binding protein Rit1	RIT1	2.49	2.35	7.59 × 10^−4^
E3 ubiquitin-protein ligase RNF114	RNF114	2.35	2.30	2.13 × 10^−4^
RWD domain-containing protein 1	RWDD1	2.34	2.03	1.63 × 10^−4^
Deoxynucleoside triphosphate triphosphohydrolase SAMHD1	SAMHD1	8.28	6.54	9.29 × 10^−8^
GTP-binding protein SAR1a	SAR1A	2.61	2.33	1.37 × 10^−5^
Sodium-coupled neutral amino acid transporter 2	SLC38A2	2.17	2.29	1.12 × 10^−4^
Protein sprouty homolog 4	SPRY4	3.37	3.30	7.71 × 10^−6^
Signal transducer and activator of transcription 2	STAT2	2.49	2.21	1.12 × 10^−5^
Antigen peptide transporter 2	TAP2	2.04	2.09	1.80 × 10^−6^
Tax1-binding protein 1	TAX1BP1	5.42	5.74	2.15 × 10^−9^
Tumor necrosis factor receptor superfamily member 10A	TNFRSF10A	3.48	3.75	1.50 × 10^−6^
Tripartite motif-containing protein 14	TRIM14	2.18	2.10	2.27 × 10^−6^
E3 ubiquitin-protein ligase TRIM38	TRIM38	10.57	11.20	1.19 × 10^−6^
TSC22 domain family protein 2	TSC22D2	2.24	2.37	5.14 × 10^−5^
Thioredoxin-like protein 1	TXNL1	2.65	2.24	1.90 × 10^−6^
tRNA wybutosine-synthesizing protein 3 homolog	TYW3	2.55	2.24	1.14 × 10^−4^
Ubiquitin-associated and SH3 domain-containing protein B	UBASH3B	2.26	2.15	4.69 × 10^−4^
Ubiquitin/ISG15-conjugating enzyme E2 L6	UBE2L6	4.59	3.82	2.30 × 10^−6^
Ubiquitin thioesterase OTU1	YOD1	9.71	7.68	1.37 × 10^−4^
YrdC domain-containing protein, mitochondrial	YRDC	2.52	2.43	7.59 × 10^−8^

## Data Availability

All relevant data are within the paper.

## Supplementary Materials

The following supporting information can be downloaded at: https://www.mdpi.com/article/10.3390/jpm14030330/s1, Figure S1: Original image of agarose gel electrophoresis. A, original image of Figure 2B; B, original image of Figure 2C.
