# Supplementary material for: Synthetic Circular RNA for microRNA-1269a Suppresses Tumor Progression in Oral Squamous Cell Carcinoma
Source: Cancers (Basel). 2024 Mar 21;16(6):1242. doi: 10.3390/cancers16061242 (PMC10969775; doi:10.3390/cancers16061242)
Supplement: Supplementary file 1 [file cancers-16-01242-s001.zip › cancers-2891777-supplementary.pdf]

Supplementary Materials:

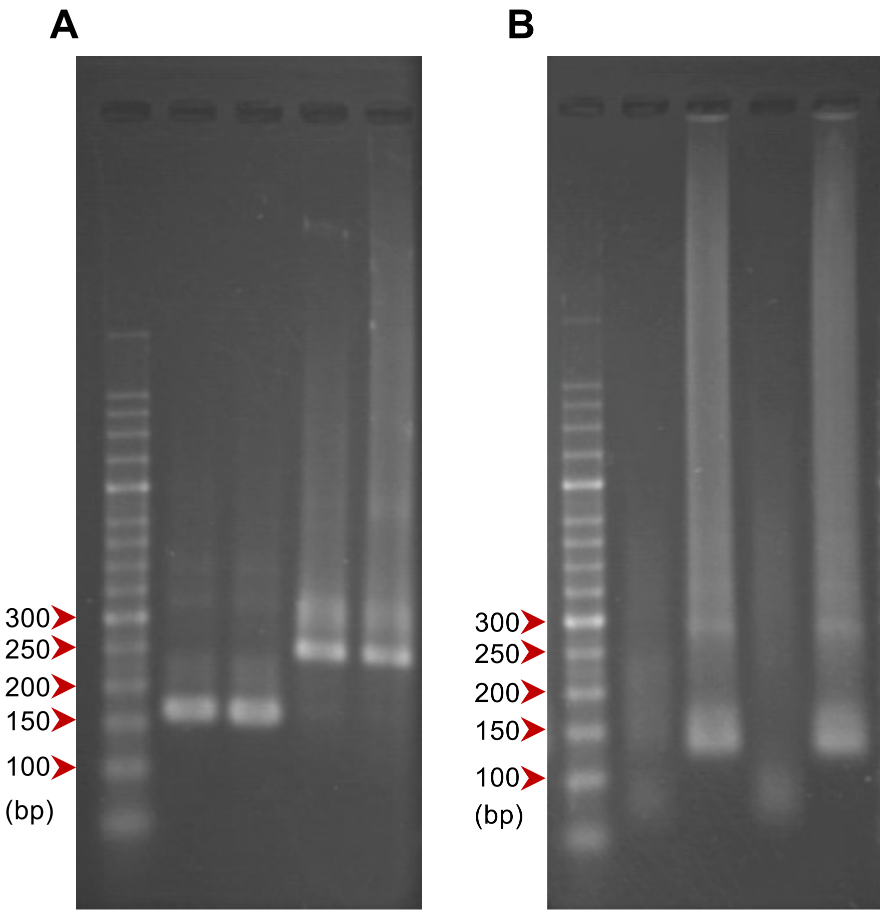

Figure S1: Original image of agarose gel electrophoresis. (A) Original image of Figure 2B; (B) Original image of Figure 2C.
